# Pituitary Adenylate Cyclase-Activating Polypeptide Alleviates Intestinal, Extra-Intestinal and Systemic Inflammatory Responses during Acute *Campylobacter jejuni*-induced Enterocolitis in Mice

**DOI:** 10.3390/pathogens9100805

**Published:** 2020-09-30

**Authors:** Markus M. Heimesaat, Soraya Mousavi, Sigri Kløve, Claudia Genger, Dennis Weschka, Andrea Tamas, Dora Reglodi, Stefan Bereswill

**Affiliations:** 1 Institute of Microbiology, Infectious Diseases and Immunology, Charité-University Medicine Berlin, Corporate Member of Freie Universität Berlin, Humboldt-Universität zu Berlin, and Berlin Institute of Health, 12203 Berlin, Germany; soraya.mousavi@charite.de (S.M.); sigri.klove@charite.de (S.K.); claudia.genger@charite.de (C.G.); dennis.weschka@charite.de (D.W.); stefan.bereswill@charite.de (S.B.); 2 Department of Anatomy, MTA-PTE PACAP Research Team, Centre for Neuroscience, University of Pecs Medical School, 7691 Pecs, Hungary; andreatamassz@gmail.com (A.T.); dora.reglodi@aok.pte.hu (D.R.)

**Keywords:** pituitary adenylate cyclase-activating polypeptide (PACAP), cellular protection, anti-apoptotic properties, immune modulation, anti-inflammatory effects, cell proliferation and regeneration, *Campylobacter jejuni*, acute campylobacteriosis model, host-pathogen interaction, gut-brain axis

## Abstract

Human *Campylobacter jejuni* infections are emerging, and constitute a significant health burden worldwide. The ubiquitously expressed pituitary adenylate cyclase-activating polypeptide (PACAP) is well-known for its cell-protective and immunomodulatory effects. In our actual intervention study, we used an acute campylobacteriosis model and assessed the potential disease-alleviating effects of exogenous PACAP. Therefore, secondary abiotic IL-10^−/−^ mice were perorally infected with *C. jejuni* and treated with synthetic PACAP38 intraperitoneally from day 2 until day 5 post-infection. Whereas PACAP did not interfere with the gastrointestinal colonization of the pathogen, mice from the PACAP group exhibited less severe clinical signs of *C. jejuni*-induced disease, as compared to mock controls, which were paralleled by alleviated apoptotic, but enhanced cell proliferative responses in colonic epithelia on day 6 post-infection. Furthermore, PACAP dampened the accumulation of macrophages and monocytes, but enhanced regulatory T cell responses in the colon, which were accompanied by less IFN-γ secretion in intestinal compartments in PACAP versus mock-treated mice. Remarkably, the inflammation-dampening properties of PACAP could also be observed in extra-intestinal organs, and strikingly, even the systemic circulation on day 6 post-infection. For the first time, we provide evidence that synthetic PACAP might be a promising candidate to combat acute campylobacteriosis and post-infectious sequelae.

## 1. Introduction

More than 20 years ago, the pituitary adenylate cyclase-activating polypeptide (PACAP) was identified in the hypothalamus and shown to stimulate adenylate cyclase activity in the pituitary gland [1]. The neuropeptide belongs to the vasoactive intestinal peptide (VIP)/secretin/glucagon family and exerts 68% homology to VIP. After splicing from the pre-pro-precursor, PACAP accomplishes its biological activity due to two distinct amidated forms, i.e., PACAP27 and PACAP38 [1,2]. The expression of the neuropeptide is not restricted to the nervous system, given that PACAP can be detected in peripheral organs belonging to the respiratory, endocrine, reproductive, and digestive tracts, and furthermore, in distinct parts of the immune system. Both PACAP and VIP can bind to VPAC1 and VPAC2 receptors that are abundant on immune cells, such as macrophages and lymphocytes, whereas PAC1 constitutes a PACAP specific receptor which is expressed by macrophages as opposed to lymphocytes [3,4,5]. Related to its virtual ubiquitous expression, PACAP exerts potent cell-protective, including anti-apoptotic, proliferative and regenerative as well as immunomodulatory properties [5,6]. Potent anti-inflammatory effects of exogenous PACAP have been proven in experimental autoimmune encephalomyelitis [7] and arthritis [8], for instance, whereas data regarding inflammation-dampening effects within the gastrointestinal tract are scarce. PACAP^−/−^ mice have been shown to suffer from more severe dextran sulfate sodium (DSS)-induced colitis than wildtype counterparts [9,10], but the inflammatory phenotype could be reversed upon synthetic PACAP administration [10,11]. Recent work from our group revealed that PACAP could ameliorate subacute and even acute ileitis that had been induced upon peroral application of the protozoan parasite *Toxoplasma gondii* [12,13]. To date, however, no study has been performed to address potential cell-protective and immunomodulatory effects of exogenous PACAP during acute colitis of enteropathogenic origin.

Human infections by the Gram-negative enteropathogen *Campylobacter jejuni* are progressively rising in high- and middle- as well as in low-income countries and constitute substantial health and socioeconomic burdens worldwide [14,15]. In a heterogenous reservoir of warm-blooded vertebrates, including avian species such as poultry, *C. jejuni* reside as commensals within the intestinal tract of their hosts without inducing clinical signs. Humans, however, might become infected following the ingestion of contaminated food from livestock or water sources [16,17,18]. After the gastric passage, the highly motile bacteria attach to and invade into the large intestinal epithelia and induce the recruitment of innate as well as adaptive immune cells to the intestinal mucosa and lamina propria and pronounced pro-inflammatory mediator secretion, resulting in oxidative stress, colonic epithelial apoptosis and compromised epithelial barrier function [19,20,21,22,23]. After an incubation period of 2 to 5 days, patients suffer from symptoms of varying degrees, depending on the immune status of the host on one side, and on the arsenal of virulence factors expressed by the pathogen on the other. Whereas some patients complain about rather mild symptoms such as general discomfort and subfebrile body temperature, others present with acute campylobacteriosis characterized by abdominal cramps, fever, and watery or bloody diarrhea with inflammatory mucous discharge [15,24]. In the vast majority of cases, the infected patients require—if at all—symptomatic treatment, including substitution of fluids and electrolytes, whereas antimicrobial intervention is only indicated in severe cases affecting immunocompromised patients, for instance. The course of the disease is usually self-limiting, and symptoms resolve within 14 days post-infection (p.i.) [15,24]. On rare occasions, however, post-infectious autoimmune diseases affecting the nervous system (e.g., Guillain Barré syndrome), the joints (i.e., reactive arthritis), and the intestines (e.g., celiac disease, chronic intestinal inflammation, irritable bowel syndrome) might be observed within weeks or even months after the initial infectious event [15,25,26].

Our group has recently established a murine *C. jejuni* infection and inflammation model displaying key features of acute campylobacteriosis in humans. Since (i.) conventional laboratory mice cannot be stably infected with *C. jejuni* given the physiological colonization resistance that is provided by the complex mouse-specific gut microbiota, (ii.) wildtype mice are up to 10,000 times more resistant to Toll-like Receptor-4 (TLR-4) ligands, such as lipopolysaccharide (LPS) and lipooligosaccharide (LOS) constituting integral components of the Gram-negative bacterial cell wall, as compared to human [27], and (iii.) interleukin (IL)-10 gene deficiency renders mice susceptible to both LPS and LOS [28], we generated microbiota-depleted IL-10^−/−^ mice upon broad-spectrum antibiotic treatment. Within one week following oral *C. jejuni* infection, secondary abiotic IL-10^−/−^ mice developed acute enterocolitis characterized by wasting and bloody diarrhea [29]. These acute inflammatory responses were not restricted to the intestinal tract, but could also be observed in extra-intestinal organs and even in systemic compartments, and were furthermore all induced by *C. jejuni* LOS and mediated by activated TLR-4 dependent signaling [28,29]. Within the frame of our very recent murine intervention studies, we have reliably tested distinct vitamins, including ascorbate [30] and vitamin D [31], and plant-derived dietary compounds such as carvacrol [32] for their cytoprotective and anti-inflammatory properties in the acute campylobacteriosis model so far.

In our actual preclinical intervention study, we surveyed the potential cell-protective and immunomodulatory effects of exogenous PACAP during acute colitis of enteropathogenic origin, for the very first time, applying *C. jejuni* infected secondary abiotic IL-10^−/−^ mice.

## 2. Results

### 2.1. Pathogenic Establishment within the Gastrointestinal Tract Following PACAP Treatment of C. jejuni Infected Secondary Abiotic IL-10^−/−^ Mice

**Figure 1:**
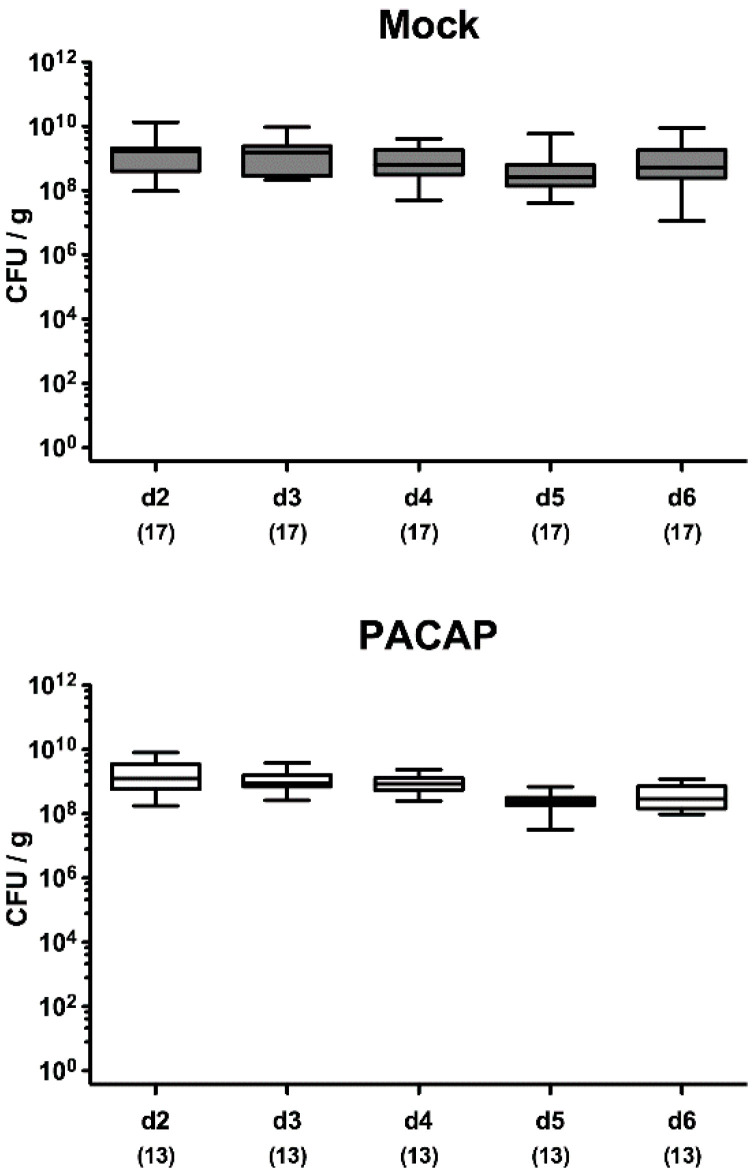
Time course of fecal pathogen loads following pituitary adenylate cyclase-activating polypeptide (PACAP) treatment of *C. jejuni* infected secondary abiotic IL-10^−/−^ mice. Mice were perorally infected with *C. jejuni* strain 81–176 by gavage on day (d) 0 and d1 and subjected to intraperitoneal treatment with either synthetic PACAP (lower panel) or vehicle (mock; upper panel), from d2 until d5 post-infection. Fecal pathogen loads were quantitated daily until d6 post-infection by culture (in colony forming units per g; CFU/g). Box plots indicate the 75th and 25th percentiles of the median (black bar within boxes). The total range and total numbers of mice under investigation (in parentheses) are indicated. Results pooled from four independent experiments are shown.

**Figure 2:**
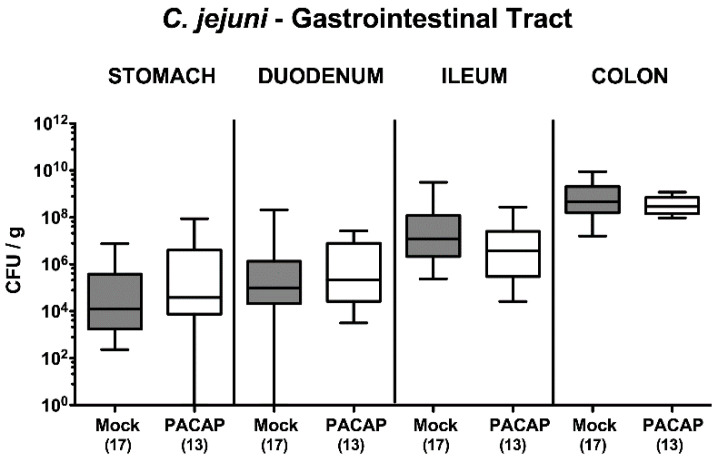
Gastrointestinal pathogen loads following PACAP treatment of *C. jejuni* infected secondary abiotic IL-10^−/−^ mice. Mice were orally infected with *C. jejuni* strain 81–176 on day (d) 0 and d1 and subjected to intraperitoneal treatment with either synthetic PACAP or vehicle (mock), from d2 until d5 post-infection. On d6, *C. jejuni* numbers were enumerated in defined compartments of the gastrointestinal tract as indicated (in colony forming units per g; CFU/g). Box plots indicate the 75th and 25th percentiles of the median (black bar within boxes). The total range and the total numbers of mice under investigation (in parentheses) are indicated. Results pooled from four independent experiments are shown.

On two consecutive days (i.e., days 0 and 1), secondary abiotic IL-10^−/−^ mice were perorally infected with 10^9^ colony forming units (CFU) *C. jejuni* strain 81–176 by gavage. From day 2 until day 5 p.i., infected mice were either treated with synthetic PACAP38 or subjected to vehicle (mock cohort) via the intraperitoneal route. Cultural analyses of fecal samples over time revealed that the pathogen could effectively colonize the intestines of either cohort at comparable median loads of approximately 10^9^ viable *C. jejuni* cells over time following infection (Figure 1). Upon necropsy on day 6 p.i., both PACAP and mock-treated mice harbored comparable pathogen burdens in distinct luminal parts of the gastrointestinal tract, as shown for the stomach, duodenum, ileum and colon (Figure 2). Hence, PACAP treatment did not interfere with the gastrointestinal colonization capabilities of the pathogen.

### 2.2. Clinical Signs Following PACAP Treatment of C. jejuni Infected Secondary Abiotic IL-10^−/−^ Mice

**Figure 3:**
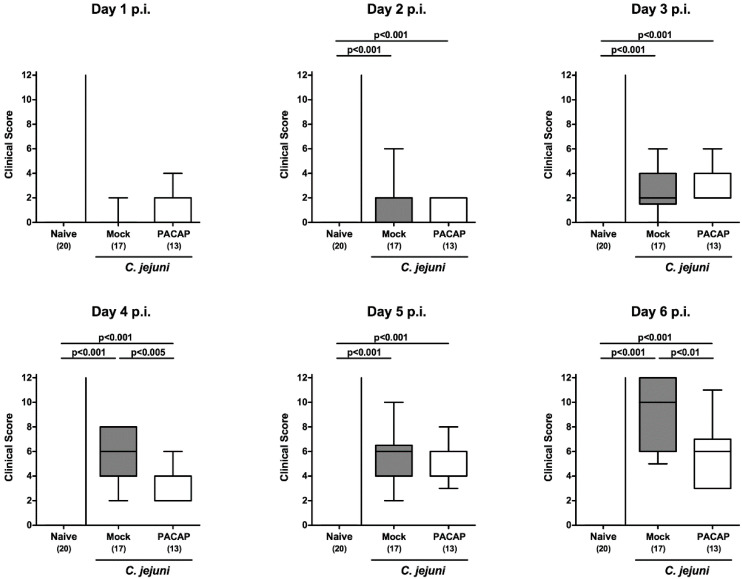
Clinical course following PACAP treatment of *C. jejuni* infected secondary abiotic IL-10^−/−^ mice. Mice were orally infected with *C. jejuni* strain 81–176 on day (d) 0 and d1 and subjected to intraperitoneal treatment, with either synthetic PACAP or vehicle (mock) from d2 until d5 post-infection. The clinical signs were quantitated daily according to defined clinical scores. Naive mice were used as negative controls. Box plots indicate the 75th and 25th percentiles of the median (black bar within boxes). The total range, the significance levels (*p* values calculated by the one-way ANOVA test followed by the Tukey post-correction test for multiple comparisons) and the total numbers of mice under investigation (in parentheses) are indicated. Results pooled from four independent experiments are shown.

By using a standardized clinical scoring system assessing typical clinical features of acute human campylobacteriosis such as wasting and bloody diarrhea, we further quantitatively surveyed the clinical conditions of infected mice from either treatment regimen on a daily basis. As early as day 4 p.i., PACAP treated mice displayed lower clinical scores versus mock-treated control mice (*p* < 0.005; Figure 3). This also held true for the day of necropsy (*p* < 0.01; Figure 3), given that on day 6 p.i., mice from the PACAP cohort suffered less distinctly from wasting and diarrhea in particular when compared to mock controls (*p* < 0.001 and *p* < 0.005, respectively; Figure S1A,C), whereas the abundance of fecal blood could be observed to a similar extent (not significant (n.s.); Figure S1B). Hence, PACAP treatment resulted in less severe *C. jejuni*-induced disease.

### 2.3. Apoptotic and Proliferating Cell Responses in Colonic Epithelia Following PACAP Treatment of C. jejuni Infected Secondary Abiotic IL-10^−/−^ Mice

**Figure 4:**
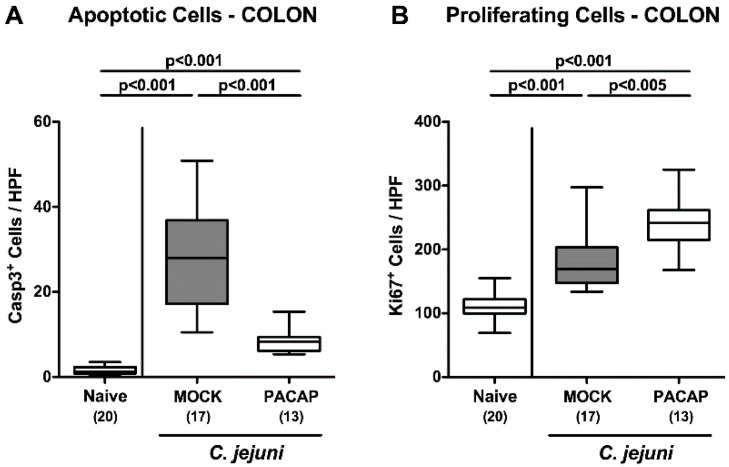
Apoptotic and proliferating colonic epithelial cells following PACAP treatment of *C. jejuni* infected secondary abiotic IL-10^−/−^ mice. Mice were perorally infected with *C. jejuni* strain 81–176 by gavage on day (d) 0 and d1, and subjected to intraperitoneal treatment, with either synthetic PACAP or vehicle (mock) from d2 until d5 post-infection. On d6, the average numbers of (**A**) apoptotic (cleaved caspase-3 positive, Casp3^+^) and (**B**) proliferating (Ki67^+^) epithelial cells were quantitated in six high power fields (HPF) of colonic paraffin sections applying immunohistochemistry. Naive mice were used as negative controls. Box plots represent the 75th and 25th percentiles of the median (black bar inside the boxes). The total range, the significance levels (*p* values calculated by the one-way ANOVA test followed by the Tukey post-correction test for multiple comparisons) and the total numbers of mice under investigation (in parentheses) are indicated. Results pooled from four independent experiments are shown.

Since intestinal apoptosis constitutes a reliable parameter for the intestinal inflammatory grading [33], we stained colonic paraffin sections with an antibody directed against cleaved caspase-3. Our immunohistochemical analyses revealed that *C. jejuni* infection resulted in marked increases in caspase-3^+^ colonic epithelial cells (*p* < 0.001; Figure 4A). These apoptotic responses were, however, far less pronounced following PACAP as compared to mock treatment (*p* < 0.001; Figure 4A). Conversely, numbers of Ki67^+^ cells in colonic epithelia indicative for cell proliferation were higher on day 6 p.i. as compared to naive conditions (*p* < 0.001), which was also the case upon PACAP versus mock application (*p* < 0.005; Figure 4B). Hence, PACAP treatment alleviated apoptotic, but enhanced cell proliferative responses in colonic epithelia thereby antagonizing mucosal cell damage upon pathogenic infection.

### 2.4. Large Intestinal Innate and Adaptive Immune Cell Responses Following PACAP Treatment of C. jejuni Infected Secondary Abiotic IL-10^−/−^ Mice

**Figure 5:**
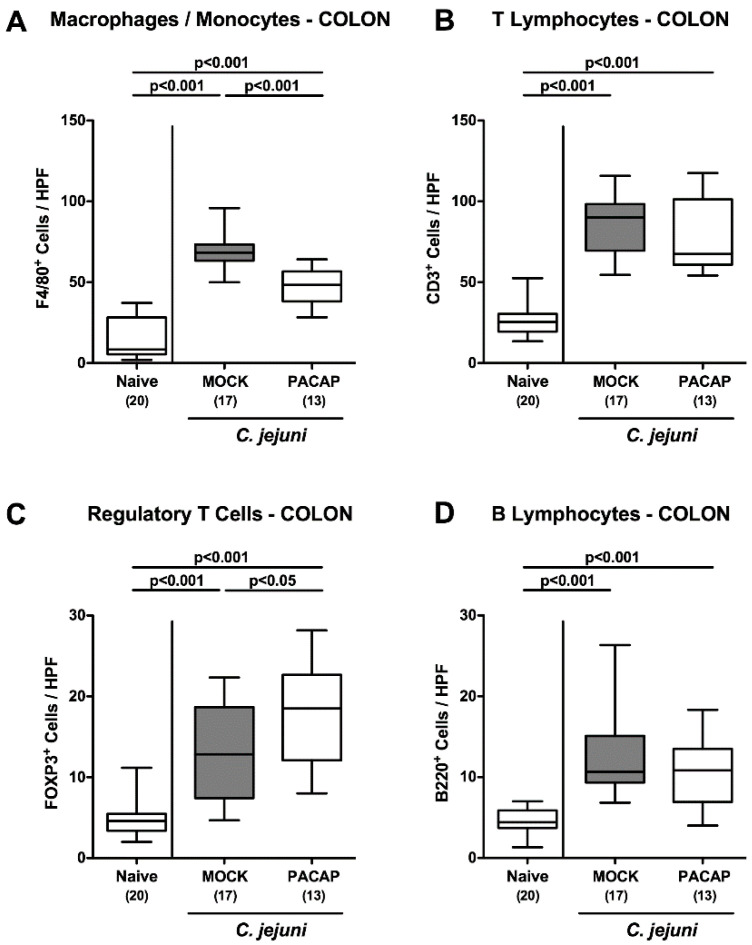
Innate and adaptive immune cell responses in the colon following PACAP treatment of *C. jejuni* infected secondary abiotic IL-10^−/−^ mice. Mice were perorally infected with *C. jejuni* strain 81–176 by gavage on day (d) 0 and d1, and subjected to intraperitoneal treatment with either synthetic PACAP or vehicle (mock) from d2 until d5 post-infection. On d6 the average numbers of (**A**) macrophages and monocytes (F4/80^+^), (**B**) T lymphocytes (CD3^+^), (**C**) regulatory T cells (FOXP3^+^) and (**D**) B lymphocytes (B220^+^) were quantitated in six high power fields (HPF) of colonic paraffin sections applying immunohistochemistry. Naive mice were used as negative controls. Box plots indicate the 75th and 25th percentiles of the median (black bar within boxes). The total range, the significance levels (*p* values calculated by the one-way ANOVA test followed by the Tukey post-correction test for multiple comparisons) and the total numbers of mice under investigation (in parentheses) are indicated. Results pooled from four independent experiments are shown.

Next, we surveyed the immunomodulatory effects of PACAP in acute *C. jejuni*-induced enterocolitis. Therefore, distinct cell populations of innate and adaptive immunity were quantitated following the immunohistochemical staining of colonic ex vivo biopsies. Six days after *C. jejuni* infection increased F4/80^+^ cell counts indicative of macrophages and monocytes could be determined in the large intestinal mucosa and lamina propria (*p* < 0.001), but with lower numbers in PACAP treated versus mock control mice (*p* < 0.001; Figure 5A). In line with the pathogen-induced innate immune cell responses, adaptive immune cell subsets such as CD3^+^ lymphocytes, FOXP3^+^ regulatory T cells and B220^+^ B lymphocytes increased during *C. jejuni* infection (*p* < 0.001; Figure 5B–D), whereas even higher colonic regulatory T cell counts could be assessed in PACAP versus mock-treated mice on day 6 p.i. (*p* < 0.05; Figure 5C). Hence, PACAP dampened pathogen-induced colonic accumulation of innate immune cells such as macrophages and monocytes, but enhanced regulatory T cell responses in the colonic mucosa and lamina propria.

### 2.5. Intestinal IFN-γ Secretion Following PACAP Treatment of C. jejuni Infected Secondary Abiotic IL-10^−/−^ Mice

**Figure 6:**
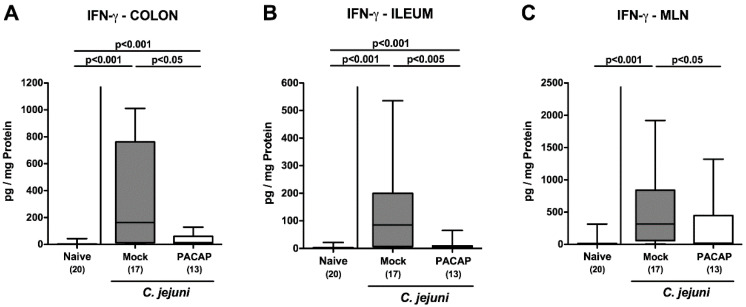
Intestinal IFN-γ secretion following PACAP treatment of *C. jejuni* infected secondary abiotic IL-10^−/−^ mice. Mice were perorally infected with *C. jejuni* strain 81–176 by gavage on day (d) 0 and d1, and subjected to intraperitoneal treatment with either synthetic PACAP or vehicle (mock) from d2 until d5 post-infection. On d6, IFN-γ concentrations were measured in ex vivo biopsies derived from the (**A**) colon, (**B**) ileum and (**C**) mesenteric lymph nodes (MLN). Naive mice were used as negative controls. Box plots indicate the 75th and 25th percentiles of the median (black bar within boxes). The total range, the significance levels (*p* values calculated by the one-way ANOVA test, followed by the Tukey post-correction test for multiple comparisons) and the total numbers of mice under investigation (in parentheses) are indicated. Results pooled from four independent experiments are shown.

Next, we assessed the pro-inflammatory interferon (IFN)-γ secretion in the intestinal tract. Increased IFN-γ concentrations were measured in colonic ex vivo explant supernatants on day 6 following *C. jejuni* infection (*p* < 0.001), whereas pro-inflammatory cytokine secretion was less pronounced in PACAP as compared to mock-treated mice (*p* < 0.05; Figure 6A). Remarkably, the pathogen additionally induced an enhanced IFN-γ secretion in the ileum and the mesenteric lymph nodes (MLN) draining the infected intestines of mock-treated mice only (*p* < 0.001), whereas IFN-γ secretion was comparable in respective intestinal sites of naive and PACAP treated mice on day 6 p.i. (Figure 6B,C). Thus, PACAP treatment dampened IFN-γ secretion alongside the intestinal tract.

### 2.6. Extra-Intestinal Pro-Inflammatory Mediator Secretion Following PACAP Treatment of C. jejuni Infected Secondary Abiotic IL-10^−/−^ Mice

**Figure 7:**
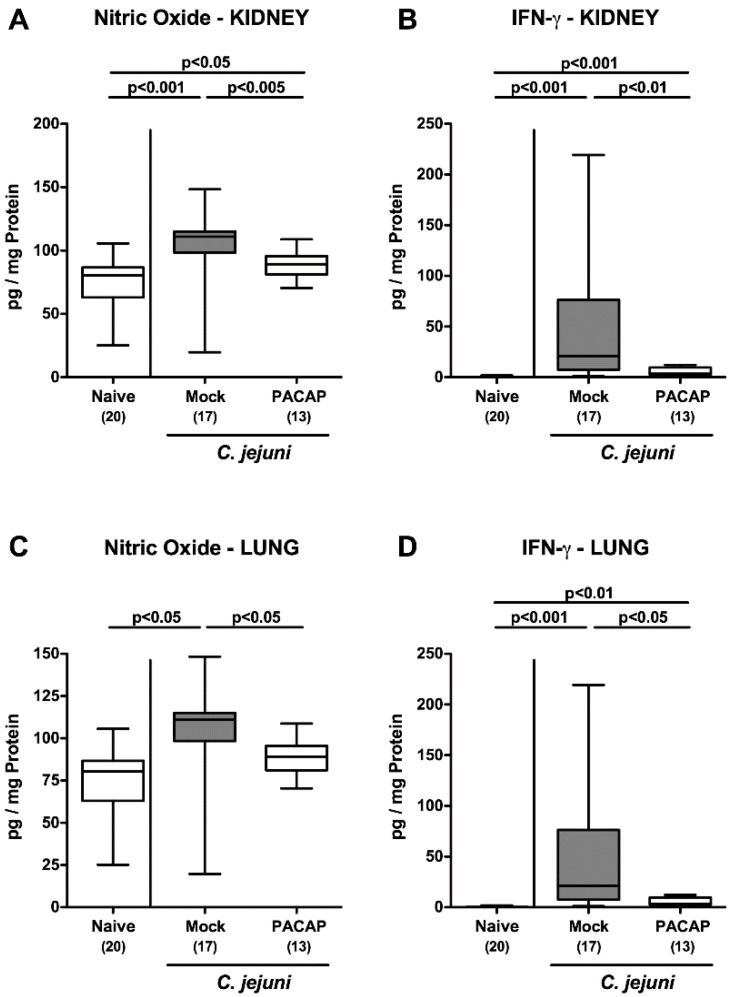
Extra-intestinal pro-inflammatory mediator secretion following PACAP treatment of *C. jejuni* infected secondary abiotic IL-10^−/−^ mice. Mice were perorally infected with *C. jejuni* strain 81–176 by gavage on day (d) 0 and d1 and subjected to intraperitoneal treatment with either synthetic PACAP or vehicle (mock) from d2 until d5 post-infection. On d6, nitric oxide (**A**,**C**) and IFN-γ (**B**,**D**) concentrations were measured in ex vivo biopsies derived from the kidney (**A**,**B**) and lung (**C**,**D**). Naive mice were used as negative controls. Box plots indicate the 75th and 25th percentiles of the median (black bar within boxes). The total range, the significance levels (*p* values calculated by the one-way ANOVA test followed by the Tukey post-correction test for multiple comparisons) and the total numbers of mice under investigation (in parentheses) are indicated. Results pooled from four independent experiments are shown.

Next, we were wondering whether the inflammation-dampening effect of exogenous PACAP was restricted to the intestinal tract or could even be observed in extra-intestinal sites. Therefore, we measured pro-inflammatory mediators in supernatants of cultured ex vivo biopsies derived from kidneys and lungs. On day 6 p.i., elevated nitric oxide and IFN-γ concentrations were detected in the kidneys of mock and PACAP treated mice (*p* < 0.05–0.001; Figure 7A,B), but with lower concentrations in the latter as compared to the former (*p* < 0.01–0.005; Figure 7A,B), which also held true for pulmonal IFN-γ concentrations (Figure 7D). Remarkably, *C. jejuni* infection enhanced nitric oxide secretion in lungs taken from mock (*p* < 0.05; Figure 7C), as opposed to PACAP treated mice (n.s. versus naive; Figure 7C). Hence, the inflammation-dampening effects of exogenous PACAP were not restricted to the intestinal tract, but also effective in extra-intestinal organs, including the kidneys and the lungs.

### 2.7. Systemic Pro-Inflammatory Cytokine Secretion Following PACAP Treatment of C. jejuni Infected Secondary Abiotic IL-10^−/−^ Mice

**Figure 8:**
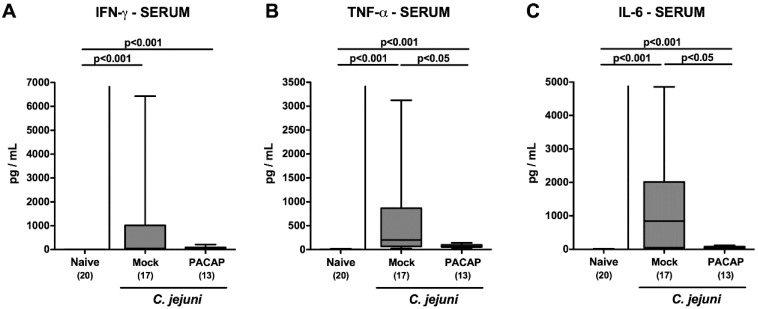
Systemic pro-inflammatory cytokine secretion following PACAP treatment of *C. jejuni* infected secondary abiotic IL-10^−/−^ mice. Mice were perorally infected with *C. jejuni* strain 81–176 by gavage on day (d) 0 and d1 and subjected to intraperitoneal treatment with either synthetic PACAP or vehicle (mock) from d2 until d5 post-infection. On d6 (**A**) IFN-γ, (**B**) TNF-α and (**C**) IL-6 concentrations were measured in serum samples. Naive mice were used as negative controls. Box plots indicate the 75th and 25th percentiles of the median (black bar within boxes). The total range, the significance levels (*p* values calculated by the one-way ANOVA test followed by the Tukey post-correction test for multiple comparisons) and the total numbers of mice under investigation (in parentheses) are indicated. Results pooled from four independent experiments are shown.

Finally, we addressed whether PACAP could even alleviate systemic immune responses during acute *C. jejuni*-induced enterocolitis. In fact, *C. jejuni* infection resulted in enhanced IFN-γ, tumor necrosis factor (TNF)-α and IL-6 secretion into the serum (*p* < 0.001; Figure 8). In the case of TNF-α and IL-6, however, these increases were less pronounced following PACAP as compared to mock treatment on day 6 p.i. (*p* < 0.05; Figure 8B,C). Hence, exogenous PACAP could effectively exert its inflammation-dampening effects even systemically in acute *C. jejuni-induced* enterocolitis.

## 3. Discussion

The cell-protective effects of PACAP in the central as well as the peripheral nervous system are mainly due to its anti-apoptotic and immunomodulatory properties. The fact that PACAP is expressed almost everywhere in the vertebrate host suggests that the pleiotropic regulatory and cytoprotective effects of the neuropeptide are not limited to the nervous system, but might additionally be effective in other tissue sites, such as the intestinal tract. In fact, our previous studies revealed that application of synthetic PACAP38 could effectively ameliorate intestinal inflammatory conditions, as shown in both subacute and even acute murine ileitis [12,13].

For the first time, our present preclinical intervention study highlights that the anti-apoptotic and immunomodulatory properties of exogenous PACAP were also effective in an acute intestinal inflammation model of different (namely infectious, enteropathogenic) etiology, and additionally, in a different affected intestinal compartment such as the large intestinal tract. PACAP treatment did not interfere with the colonization properties of the pathogen within the gastrointestinal tract, as shown by comparable luminal *C. jejuni* loads in stomach, duodenum, ileum and colon in mice of either treatment regimen. This result is further underlined by our previous in vitro study, in which we could exclude any antibacterial effects by the working solutions of both, the synthetic PACAP and the vehicle solution [12,13]. Upon PACAP treatment starting two days after the induction of acute enterocolitis, however, mice were less distinctly suffering from *C. jejuni*-induced clinical signs such as wasting and diarrhea. The better clinical outcome upon exogenous PACAP could also be observed in subacute and even acute non-self-limiting murine ileitis [12,13]. In support, PACAP^−/−^ mice were more compromised during acute DSS colitis as compared to wildtype control animals [9,10], whereas the intraperitoneal application of synthetic PACAP could sufficiently reverse the inflammatory phenotype [9].

In our present study, PACAP treatment did not only result in a better macroscopic, clinical outcome of mice, but also in less severe inflammatory sequelae on the microscopic level, as indicated by dampened apoptotic cell responses in large intestinal epithelia that were accompanied by enhanced colonic cell-proliferative and regenerative measures, antagonizing *C. jejuni*-induced cell damage. The potent immunomodulatory properties of PACAP could also be shown in our actual preclinical intervention study. For instance, PACAP treatment could dampen enteropathogen-induced colonic accumulation of innate immune cell populations (i.e., macrophages and monocytes), which was paralleled by decreased IFN-γ secretion in distinct intestinal compartments, including the colon, ileum and MLN. In support, PACAP has been shown to act as a “macrophages inactivating factor” [34], resulting in less pro-inflammatory cytokine release by stimulated macrophages upon coincubation with synthetic PACAP [35]. In our previous work, lower numbers of recruited innate immune cells could be assessed within the small intestinal mucosa and lamina propria of PACAP versus mock-treated mice with acute ileitis, which in turn led to a diminished oxidative stress to the ileal epithelia [12]. In support, a reduced influx of innate immune cells into the peritoneal cavity could be shown in PACAP treated mice with acute peritonitis [36].

Interestingly, in the present study, PACAP enhanced regulatory T cell responses in the colonic mucosa and lamina propria during acute enterocolitis, which was also the case during acute ileitis, given that higher FOXP3^+^ cell numbers could be assessed in the terminal ileum of PACAP as compared to mock-treated mice [12]. These results are further supported by studies in experimental autoimmune encephalomyelitis, showing reduced proliferating regulatory T cell numbers in PACAP^−/−^ as compared to wildtype mice [37,38]. Of note, PACAP treatment resulted in the generation of tolerogenic dendritic cells (DCs), which subsequently induce functional regulatory T cells contributing to the maintenance of peripheral tolerance [39,40,41]. It is therefore tempting to speculate that the enhanced recruitment of colonic FOXP3^+^ cells upon PACAP treatment of *C. jejuni* infected mice might have been a result of the PACAP-induced “tolerogenic DC—regulatory T cell axis” as one of the mechanisms counteracting enteropathogenic infection and induced enterocolitis.

Furthermore, PACAP treatment of *C. jejuni* infected mice dampened the secretion of the pro-inflammatory cytokine IFN-γ in distinct compartments of the intestinal tract such as the the colon, ileum and MLN during acute enterocolitis, which was also the case during acute *T. gondii-induced* ileitis [12]. In line, PACAP was shown to inhibit the expression of T helper cell-1 (Th1) type cytokines in primed CD4^+^ T lymphocytes in vitro, whereas lower numbers of IFN-γ secreting cells could be detected in antigen-immunized mice that had been subjected to exogenous PACAP [42]. Remarkably, the inflammation-dampening properties of PACAP were not restricted to the intestinal tract, but also effective in extra-intestinal tissue sites as indicated by lower IFN-γ and nitric oxide concentrations in kidneys and lungs, resulting in less oxidative stress to respective organs. In line, PACAP treatment could ameliorate histopathological sequelae in both lungs and kidneys of *T. gondii* infected mice suffering from acute ileitis [12], whereas lower pulmonal IFN-γ concentrations were assessed in ex vivo biopsies derived from PACAP versus mock-treated mice with subacute ileitis [13]. Furthermore, PACAP treatment resulted in pronounced cell protective effects in several models of renal injury [43,44,45,46,47,48,49,50,51], which also held true for acute endotoxin-induced inflammation of the lung [52]. Due to their potent broncho-relaxant as well as anti-inflammatory modes of actions, synthetic PACAP analogues have been approved as therapeutic options against bronchial asthma [52,53].

Strikingly, in our actual intervention study, the disease-alleviating features of exogenous PACAP were also effective systemically as indicated by lower TNF-α and IL-6 concentration in serum samples obtained from PACAP, as compared to mock-treated mice suffering from acute *C. jejuni*-induced enterocolitis. These results are well supported by our previous studies, were PACAP treatment decreased splenic TNF-α and serum IL-6 concentrations during subacute *T. gondii*-induced ileitis [13]. Systemic anti-inflammatory effects of PACAP were further demonstrated in previous studies, showing that PACAP treatment provided protection from experimental endotoxin-induced sepsis and shock [43,54,55]. In the acute enterocolitis model applied here, *C. jejuni*-induced disease has been shown to be mainly due to TLR-4 dependent signaling of bacterial LOS [28], which is also the case in subacute and even acute *T. gondii*-induced ileitis, where the inflammatory conditions are initiated and accelerated by TLR-4 ligands derived from the commensal gut microbiota overgrowing the inflamed intestinal tract, which results in dysbiosis [13,56,57,58]. Furthermore, it is highly likely that, despite the absence of intact, viable *C. jejuni* cells in kidneys, lungs and blood (not shown), pathogenic LOS was responsible for increased pro-inflammatory mediator secretion in respective compartments, which could be decreased upon PACAP administration to infected mice. In line with the disease-alleviating effects of PACAP during endotoxin-induced pulmonary [52] and systemic inflammation [43,54,55], and given that PACAP could effectively counteract TLR-4 activation in traumatic brain injury [59], the dampening of the TLR-4 dependent signaling cascade appears to be one of the key events underlying the pleiotropic health-beneficial effects of the compound.

It is noteworthy that, in our actual invention study, PACAP had been applied to *C. jejuni*-infected mice on four consecutive days only. Since our previous work revealed time-of-treatment dependent anti-inflammatory properties of the exogenous PACAP38 during acute *T. gondii* ileitis [12], it is highly likely that even more pronounced effects might have been achieved when applying a prophylactic treatment regimen starting before *C. jejuni* infection; this will be unraveled in future studies.

## 4. Materials and Methods

### 4.1. Ethics Statement

Mouse experiments were conducted according to the European Guidelines for Animal Welfare (2010/63/EU), following approval by the Ethical Commission for Animal Experiments headed by the “Landesamt für Gesundheit und Soziales” (LaGeSo, Berlin, registration number G0104/19; approval date: 15 July 2019). Twice a day the clinical aspects in mice were surveyed.

### 4.2. Secondary Abiotic IL-10^−/−^ Mice

IL-10^−/−^ mice (C57BL/10 background) were bred and maintained under specific pathogen-free (SPF) conditions in the identical unit of the Forschungseinrichtungen für Experimentelle Medizin (Charité—University Medicine Berlin). Mice were kept in cages equipped with filter tops within an experimental semi-barrier under standard conditions (22–24 °C room temperature, 55 ± 15% humidity, 12 h light/12 dark cycle) with unrestricted access to autoclaved food pellets (ssniff R/M-H, V1534-300, Sniff, Soest, Germany) and tap water.

For gut microbiota depletion, 3-week-old female and male littermates were transferred into sterile cages (maximum of 4 animals per cage) and treated for eight weeks with a broad-spectrum antibiotic cocktail containing ampicillin plus sulbactam (1 g/L; Dr. Friedrich Eberth Arzneimittel, Ursensollen, Germany), vancomycin (500 mg/L; Hikma Pharmaceuticals, London, UK), ciprofloxacin (200 mg/L; Fresenius Kabi, Bad Homburg, Germany), imipenem (250 mg/L; Fresenius Kabi) and metronidazole (1 g/L; B. Braun, Melsungen, Germany) in the drinking water (ad libitum) as described earlier [33,56]. Secondary abiotic IL-10^−/−^ mice were continuously kept and handled under strict aseptic conditions and received autoclaved food and drinking water or antibiotic cocktail to minimize the risk of contaminations.

### 4.3. C. jejuni Infection, Gastrointestinal Colonization and Extra-Intestinal Translocation

For infection experiments, *C. jejuni* strain 81–176 was thawed from a stock and cultivated on columbia agar (supplemented with 5% sheep blood) and karmali agar plates (both from Oxoid, Wesel, Germany). Microbiota-depleted IL-10^−/−^ mice were infected orally with 10^9^ CFU in a volume of 0.3 mL phosphate buffered saline (PBS; Thermo Fisher Scientific, Waltham, MA, USA) by gavage on days 0 and 1, as stated earlier [33]. For a kinetic assessment of the intestinal colonization efficacies of the pathogen, *C. jejuni* loads were enumerated in fecal samples every day post-infection, and in luminal samples obtained from the stomach, duodenum, ileum and colon) upon necropsy (i.e., day 6 p.i.) by culture, as described previously [33]. In brief, serial dilutions of respective samples were streaked onto columbia agar plates containing 5% sheep blood and karmali agar plates (both from Oxoid, Wesel, Germany), and incubated in a jar under microaerophilic conditions for 48 h at 37 °C.

### 4.4. Treatment with Synthetic PACAP

Synthetic PACAP38 was obtained from the Department of Medical Chemistry, University of Szeged (Hungary). Once daily mice were either subjected to synthetic PACAP (1.5 mg per kg body weight, dissolved in phosphate buffered saline (PBS, Gibco, Life Technologies, UK) or to vehicle (mock) intraperitoneally from day 2 until day 5 p.i. Any antimicrobial effects of either solution were excluded before [60,61].

### 4.5. Clinical Assessment

Prior and post pathogen infection, we quantitatively surveyed the clinical signs in mice daily applying a standardized cumulative clinical scoring system (maximum 12 points), with the following objectives: clinical aspect/wasting (0: normal; 1: ruffled fur; 2: less locomotion; 3: isolation; 4: severely compromised locomotion, pre-final aspect); fecal blood (0: no blood; 2: microscopic detection of blood by the Guajac method using Haemoccult, Beckman Coulter/PCD, Germany; 4: macroscopic blood visible), and diarrhea (0: formed feces; 2: pasty feces; 4: liquid feces), as reported previously [62].

### 4.6. Sampling Procedures

On the day of necropsy (i.e., day 6 p.i.), animals were sacrificed by CO_2_ asphyxiation. Ex vivo biopsies from MLN, kidneys, lungs, colon and ileum, as well as luminal samples from stomach, duodenum, ileum and colon, were derived under sterile conditions, whereas cardiac puncture was performed to obtain blood for serum cytokine measurements. For subsequent cultural, immunohistochemical and immunological analyses, intestinal tissue samples were collected from each mouse in parallel.

### 4.7. Immunohistochemistry

Immunohistochemical stainings were done in large intestinal ex vivo explants, following immediate fixation of the tissues in 5% formalin and embedding in paraffin as recently reported [63,64,65,66]. For enumerating apoptotic epithelial cells, 5 μm thin colonic paraffin sections were stained with primary antibodies directed against cleaved caspase 3 (Asp175, Cell Signaling, Beverly, MA, USA; 1:200); for proliferating epithelial cells against Ki67 (TEC3, Dako, Glostrup, Denmark; 1:100); for macrophages/monocytes against F4/80 (# 14-4801, clone BM8, eBioscience, San Diego, CA, USA; 1:50); for T lymphocytes against CD3 (#N1580, Dako; 1:10); for regulatory T cells (Tregs) against FOXP3 (clone FJK-165, #14-5773, eBioscience; 1:100); and for B lymphocytes against B220 (No. 14-0452-81, eBioscience; 1:200). Positively stained cells were enumerated by an independent investigator applying light microscopy. The average number of respective positively stained cells in each sample was determined within at least six high power fields (HPF, 0.287 mm^2^, 400× magnification).

### 4.8. Pro-Inflammatory Cytokines in Intestinal, Extra-Intestinal and Serum Samples

Distal large and small intestinal ex vivo explants were cut longitudinally and rinsed in PBS after which strips of approximately 1 cm^2^ were incubated in 24-flat-bottom well plates (Thermo Fisher Scientific) for 18 h at 37 °C containing 500 μL serum-free RPMI 1640 medium (Thermo Fisher Scientific) with penicillin (100 U/mL) and streptomycin (100 µg/mL; Biochrom, Berlin, Germany). Ex vivo biopsies of MLN (3 single lymph nodes), kidney (one half after longitudinal cut) and one lung were treated likewise. After incubation, respective cultured biopsy supernatants were harvested, and in addition to serum samples analyzed for IFN-γ, TNF-α, and IL-6 by the Mouse Inflammation Cytometric Bead Assay (CBA; BD Biosciences, Heidelberg, Germany) on a BD FACSCanto II flow cytometer (BD Biosciences). Nitric oxide concentrations were determined by the Griess reaction [56,67].

### 4.9. Statistical Analysis

Medians and levels of significance were determined with GraphPad Prism v8, USA. The Mann–Whitney U test was used for pairwise comparisons of not normally distributed data. For multiple comparisons, the one-sided ANOVA with Tukey post-correction was used for normally distributed data and the Kruskal–Wallis test with Dunn’s post-correction for not normally distributed data. Two-sided probability (*p*) values ≤ 0.05 were considered significant. Data were pooled from four independent experiments.

## 5. Conclusions

This is the first report showing that synthetic PACAP exerts potent intestinal, extra-intestinal and systemic disease-alleviating effects during acute *C. jejuni*-induced enterocolitis. We conclude that PACAP constitutes a promising antibiotic-independent strategy, in order to combat inflammatory responses during acute campylobacteriosis and post-infectious sequelae.

## Supplementary Materials

The following are available online at https://www.mdpi.com/2076-0817/9/10/805/s1. Figure S1: Clinical signs following PACAP treatment of *C. jejuni* infected secondary abiotic IL-10^−/−^ mice. Secondary abiotic IL-10^−/−^ mice were perorally infected with *C. jejuni* strain 81–176 by gavage on day (d) 0 and d1 and subjected to intraperitoneal treatment with either synthetic PACAP or vehicle (mock) from d2 until d5 post-infection. On d6 the clinical signs of campylobacteriosis such as (A) wasting, (B) abundance of fecal blood and (C) stool consistency were quantitated applying a clinical scoring system. Naive mice served as negative controls. Box plots represent the 75th and 25th percentiles of the median (black bar inside the boxes). The total range, the significance levels determined by the one-way ANOVA test followed by the Tukey post-correction test for multiple comparisons and the total numbers of analyzed animals (in parentheses) are indicated. Data were pooled from four independent experiments.

## Abbreviations

CBA	cytometric bead array
CFU	colony-forming units
DCs	dendritic cells
DSS	dextran sulfate sodium
HPF	high power fields
IL	interleukin
LOS	lipo-oligosaccharide
LPS	lipo-polysaccharide
MLN	mesenteric lymph nodes
n.s.	not significant
PBS	phosphate buffered saline
PACAP	pituitary adenylate cyclase-activating polypeptide
p.i.	post-infection
SPF	specific pathogen free
Th1	T helper cell-1
TLR	toll-like receptor
TNF-α	tumor necrosis factor-α
Treg	regulatory T cells
VIP	vasoactive intestinal peptide

## References

[1] Miyata A., Arimura A., Dahl R.R., Minamino N., Uehara A., Jiang L., Culler M.D., Coy D.H. (1989). Isolation of a novel 38 residue-hypothalamic polypeptide which stimulates adenylate cyclase in pituitary cells. Biochem. Biophys. Res. Commun..

[2] Vaudry D., Gonzalez B.J., Basille M., Yon L., Fournier A., Vaudry H. (2000). Pituitary adenylate cyclase-activating polypeptide and its receptors: From structure to functions. Pharmacol. Rev..

[3] Gomariz R.P., Juarranz Y., Abad C., Arranz A., Leceta J., Martinez C. (2006). VIP-PACAP system in immunity: New insights for multitarget therapy. Ann. N. Y. Acad. Sci..

[4] Abad C., Gomariz R.P., Waschek J.A. (2006). Neuropeptide mimetics and antagonists in the treatment of inflammatory disease: Focus on VIP and PACAP. Curr. Top. Med. Chem..

[5] Vaudry D., Falluel-Morel A., Bourgault S., Basille M., Burel D., Wurtz O., Fournier A., Chow B.K., Hashimoto H., Galas L. (2009). Pituitary adenylate cyclase-activating polypeptide and its receptors: 20 years after the discovery. Pharmacol. Rev..

[6] Reglodi D., Kiss P., Szabadfi K., Atlasz T., Gabriel R., Horvath G., Szakaly P., Sandor B., Lubics A., Laszlo E. (2012). PACAP is an endogenous protective factor-insights from PACAP-deficient mice. J. Mol. Neurosci..

[7] Kato H., Ito A., Kawanokuchi J., Jin S., Mizuno T., Ojika K., Ueda R., Suzumura A. (2004). Pituitary adenylate cyclase-activating polypeptide (PACAP) ameliorates experimental autoimmune encephalomyelitis by suppressing the functions of antigen presenting cells. Mult. Scler. J..

[8] Abad C., Martinez C., Leceta J., Gomariz R.P., Delgado M. (2001). Pituitary adenylate cyclase-activating polypeptide inhibits collagen-induced arthritis: An experimental immunomodulatory therapy. J. Immunol..

[9] Azuma Y.T., Hagi K., Shintani N., Kuwamura M., Nakajima H., Hashimoto H., Baba A., Takeuchi T. (2008). PACAP provides colonic protection against dextran sodium sulfate induced colitis. J. Cell. Physiol..

[10] Nemetz N., Abad C., Lawson G., Nobuta H., Chhith S., Duong L., Tse G., Braun J., Waschek J.A. (2008). Induction of colitis and rapid development of colorectal tumors in mice deficient in the neuropeptide PACAP. Int. J. Cancer.

[11] Abad C., Martinez C., Juarranz M.G., Arranz A., Leceta J., Delgado M., Gomariz R.P. (2003). Therapeutic effects of vasoactive intestinal peptide in the trinitrobenzene sulfonic acid mice model of Crohn’s disease. Gastroenterology.

[12] Heimesaat M.M., Dunay I.R., Schulze S., Fischer A., Grundmann U., Alutis M., Kuhl A.A., Tamas A., Toth G., Dunay M.P. (2014). Pituitary adenylate cyclase-activating polypeptide ameliorates experimental acute ileitis and extra-intestinal sequelae. PLoS ONE.

[13] Bereswill S., Escher U., Grunau A., Kuhl A.A., Dunay I.R., Tamas A., Reglodi D., Heimesaat M.M. (2019). Pituitary Adenylate Cyclase-Activating Polypeptide-A Neuropeptide as Novel Treatment Option for Subacute Ileitis in Mice Harboring a Human Gut Microbiota. Front. Immunol..

[14] Young K.T., Davis L.M., Dirita V.J. (2007). *Campylobacter jejuni*: Molecular biology and pathogenesis. Nat. Rev. Microbiol..

[15] Backert S., Tegtmeyer N., Cróinín T.Ó., Boehm M., Heimesaat M.M., Klein G. (2017). Chapter 1—Human campylobacteriosis. Campylobacter.

[16] Guerry P., Szymanski C.M. (2008). *Campylobacter* sugars sticking out. Trends Microbiol..

[17] Lane J.A., Mehra R.K., Carrington S.D., Hickey R.M. (2010). The food glycome: A source of protection against pathogen colonization in the gastrointestinal tract. Int. J. Food Microbiol..

[18] Hermans D., Pasmans F., Messens W., Martel A., Van Immerseel F., Rasschaert G., Heyndrickx M., Van Deun K., Haesebrouck F. (2012). Poultry as a host for the zoonotic pathogen *Campylobacter jejuni*. Vector Borne Zoonotic Dis..

[19] van Spreeuwel J.P., Duursma G.C., Meijer C.J., Bax R., Rosekrans P.C., Lindeman J. (1985). *Campylobacter* colitis: Histological immunohistochemical and ultrastructural findings. Gut.

[20] Walker R.I., Caldwell M.B., Lee E.C., Guerry P., Trust T.J., Ruiz-Palacios G.M. (1986). Pathophysiology of *Campylobacter* enteritis. Microbiol. Rev..

[21] Janssen R., Krogfelt K.A., Cawthraw S.A., van Pelt W., Wagenaar J.A., Owen R.J. (2008). Host-pathogen interactions in *Campylobacter* infections: The host perspective. Clin. Microbiol. Rev..

[22] Havelaar A.H., van Pelt W., Ang C.W., Wagenaar J.A., van Putten J.P., Gross U., Newell D.G. (2009). Immunity to *Campylobacter*: Its role in risk assessment and epidemiology. Crit. Rev. Microbiol..

[23] Ó Cróinín T., Backert S. (2012). Host epithelial cell invasion by *Campylobacter jejuni: Trigger* or zipper mechanism?. Front. Cell. Infect. Microbiol..

[24] Kist M., Bereswill S. (2001). Campylobacter jejuni. Contrib. Microbiol..

[25] Allos B.M. (1997). Association between *Campylobacter* infection and Guillain-Barre syndrome. J. Infect. Dis..

[26] Mortensen N.P., Kuijf M.L., Ang C.W., Schiellerup P., Krogfelt K.A., Jacobs B.C., van Belkum A., Endtz H.P., Bergman M.P. (2009). Sialylation of *Campylobacter jejuni* lipo-oligosaccharides is associated with severe gastro-enteritis and reactive arthritis. Microbes Infect..

[27] Taveira da Silva A.M., Kaulbach H.C., Chuidian F.S., Lambert D.R., Suffredini A.F., Danner R.L. (1993). Brief report: Shock and multiple-organ dysfunction after self-administration of *Salmonella* endotoxin. N. Engl. J. Med..

[28] Mousavi S., Bereswill S., Heimesaat M.M. (2020). Novel Clinical *Campylobacter jejuni* Infection Models Based on Sensitization of Mice to Lipooligosaccharide, a Major Bacterial Factor Triggering Innate Immune Responses in Human Campylobacteriosis. Microorganisms.

[29] Haag L.M., Fischer A., Otto B., Plickert R., Kuhl A.A., Gobel U.B., Bereswill S., Heimesaat M.M. (2012). *Campylobacter jejuni* induces acute enterocolitis in gnotobiotic IL-10^−/−^ mice via Toll-like-receptor-2 and -4 signaling. PLoS ONE.

[30] Mousavi S., Escher U., Thunhorst E., Kittler S., Kehrenberg C., Bereswill S., Heimesaat M.M. (2020). Vitamin C alleviates acute enterocolitis in *Campylobacter jejuni* infected mice. Sci. Rep..

[31] Mousavi S., Lobo de Sa F.D., Schulzke J.D., Bucker R., Bereswill S., Heimesaat M.M. (2019). Vitamin D in Acute Campylobacteriosis-Results From an Intervention Study Applying a Clinical *Campylobacter jejuni* Induced Enterocolitis Model. Front. Immunol..

[32] Mousavi S., Schmidt A.M., Escher U., Kittler S., Kehrenberg C., Thunhorst E., Bereswill S., Heimesaat M.M. (2020). Carvacrol ameliorates acute campylobacteriosis in a clinical murine infection model. Gut Pathog..

[33] Bereswill S., Fischer A., Plickert R., Haag L.M., Otto B., Kuhl A.A., Dasti J.I., Zautner A.E., Munoz M., Loddenkemper C. (2011). Novel murine infection models provide deep insights into the “menage a trois” of *Campylobacter jejuni*, microbiota and host innate immunity. PLoS ONE.

[34] Ganea D., Delgado M. (2002). Vasoactive intestinal peptide (VIP) and pituitary adenylate cyclase-activating polypeptide (PACAP) as modulators of both innate and adaptive immunity. Crit. Rev. Oral Biol. Med..

[35] Laskin D.L., Pendino K.J. (1995). Macrophages and inflammatory mediators in tissue injury. Annu. Rev. Pharmacol. Toxicol..

[36] Delgado M., Ganea D. (2001). Inhibition of endotoxin-induced macrophage chemokine production by vasoactive intestinal peptide and pituitary adenylate cyclase-activating polypeptide in vitro and in vivo. J. Immunol..

[37] Tan Y.-V., Abad C., Lopez R., Dong H., Liu S., Lee A., Gomariz R.P., Leceta J., Waschek J.A. (2009). Pituitary adenylyl cyclase-activating polypeptide is an intrinsic regulator of Treg abundance and protects against experimental autoimmune encephalomyelitis. Proc. Natl. Acad. Sci. USA.

[38] Tan Y.V., Abad C., Wang Y., Lopez R., Waschek J.A. (2013). Pituitary adenylate cyclase activating peptide deficient mice exhibit impaired thymic and extrathymic regulatory T cell proliferation during EAE. PLoS ONE.

[39] Delgado M. (2009). Generating tolerogenic dendritic cells with neuropeptides. Hum. Immunol..

[40] Delgado M., Gonzalez-Rey E., Ganea D. (2006). Vasoactive intestinal peptide: The dendritic cell --> regulatory T cell axis. Ann. N. Y. Acad. Sci..

[41] Delgado M., Gonzalez-Rey E., Ganea D. (2005). The neuropeptide vasoactive intestinal peptide generates tolerogenic dendritic cells. J. Immunol..

[42] Delgado M., Leceta J., Gomariz R.P., Ganea D. (1999). Vasoactive intestinal peptide and pituitary adenylate cyclase-activating polypeptide stimulate the induction of Th2 responses by up-regulating B7.2 expression. J. Immunol..

[43] Delgado M., Abad C., Martinez C., Juarranz M.G., Leceta J., Ganea D., Gomariz R.P. (2003). PACAP in immunity and inflammation. Ann. N. Y. Acad. Sci..

[44] Arimura A., Li M., Batuman V. (2006). Treatment of renal failure associated with multiple myeloma and other diseases by PACAP-38. Ann. N. Y. Acad. Sci..

[45] Li M., Maderdrut J.L., Lertora J.J., Arimura A., Batuman V. (2008). Renoprotection by pituitary adenylate cyclase-activating polypeptide in multiple myeloma and other kidney diseases. Regul. Pept..

[46] Khan A.M., Li M., Brant E., Maderdrut J.L., Majid D.S., Simon E.E., Batuman V. (2011). Renoprotection with pituitary adenylate cyclase-activating polypeptide in cyclosporine A-induced nephrotoxicity. J. Investig. Med..

[47] Li M., Balamuthusamy S., Khan A.M., Maderdrut J.L., Simon E.E., Batuman V. (2010). Pituitary adenylate cyclase-activating polypeptide ameliorates cisplatin-induced acute kidney injury. Peptides.

[48] Horvath G., Brubel R., Kovacs K., Reglodi D., Opper B., Ferencz A., Szakaly P., Laszlo E., Hau L., Kiss P. (2011). Effects of PACAP on oxidative stress-induced cell death in rat kidney and human hepatocyte cells. J. Mol. Neurosci..

[49] Horvath G., Opper B., Reglodi D. (2019). The neuropeptide pituitary adenylate cyclase-activating polypeptide (PACAP) is protective in inflammation and oxidative stress-induced damage in the kidney. Int. J. Mol. Sci..

[50] Sakamoto K., Kuno K., Takemoto M., He P., Ishikawa T., Onishi S., Ishibashi R., Okabe E., Shoji M., Hattori A. (2015). Pituitary adenylate cyclase-activating polypeptide protects glomerular podocytes from inflammatory injuries. J. Diabetes Res..

[51] Laszlo E., Juhasz T., Varga A., Czibere B., Kovacs K., Degrell P., Horvath G., Jancso G., Szakaly P., Tamas A. (2019). Protective Effect of PACAP on Ischemia/Reperfusion-Induced Kidney Injury of Male and Female Rats: Gender Differences. J. Mol. Neurosci..

[52] Elekes K., Sandor K., Moricz A., Kereskai L., Kemeny A., Szoke E., Perkecz A., Reglodi D., Hashimoto H., Pinter E. (2011). Pituitary adenylate cyclase-activating polypeptide plays an anti-inflammatory role in endotoxin-induced airway inflammation: In vivo study with gene-deleted mice. Peptides.

[53] Yoshihara S., Yamada Y., Abe T., Kashimoto K., Linden A., Arisaka O. (2004). Long-lasting smooth-muscle relaxation by a novel PACAP analogue in human bronchi. Regul. Pept..

[54] Delgado M., Martinez C., Pozo D., Calvo J.R., Leceta J., Ganea D., Gomariz R.P. (1999). Vasoactive intestinal peptide (VIP) and pituitary adenylate cyclase-activation polypeptide (PACAP) protect mice from lethal endotoxemia through the inhibition of TNF-alpha and IL-6. J. Immunol..

[55] Delgado M., Gomariz R.P., Martinez C., Abad C., Leceta J. (2000). Anti-inflammatory properties of the type 1 and type 2 vasoactive intestinal peptide receptors: Role in lethal endotoxic shock. Eur. J. Immunol..

[56] Heimesaat M.M., Bereswill S., Fischer A., Fuchs D., Struck D., Niebergall J., Jahn H.K., Dunay I.R., Moter A., Gescher D.M. (2006). Gram-negative bacteria aggravate murine small intestinal Th1-type immunopathology following oral infection with *Toxoplasma gondii*. J. Immunol..

[57] Heimesaat M.M., Fischer A., Jahn H.K., Niebergall J., Freudenberg M., Blaut M., Liesenfeld O., Schumann R.R., Gobel U.B., Bereswill S. (2007). Exacerbation of murine ileitis by Toll-like receptor 4 mediated sensing of lipopolysaccharide from commensal *Escherichia coli*. Gut.

[58] Erridge C., Duncan S.H., Bereswill S., Heimesaat M.M. (2010). The induction of colitis and ileitis in mice is associated with marked increases in intestinal concentrations of stimulants of TLRs 2, 4, and 5. PLoS ONE.

[59] Mao S.S., Hua R., Zhao X.P., Qin X., Sun Z.Q., Zhang Y., Wu Y.Q., Jia M.X., Cao J.L., Zhang Y.M. (2012). Exogenous administration of PACAP alleviates traumatic brain injury in rats through a mechanism involving the TLR4/MyD88/NF-kappaB pathway. J. Neurotrauma.

[60] Heimesaat M.M., Fischer A., Kuhl A.A., Gobel U.B., Gozes I., Bereswill S. (2015). Anti-Inflammatory Properties of NAP in Acute *Toxoplasma Gondii*-Induced Ileitis in Mice. Eur. J. Microbiol. Immunol..

[61] Bereswill S., Munoz M., Fischer A., Plickert R., Haag L.M., Otto B., Kuhl A.A., Loddenkemper C., Gobel U.B., Heimesaat M.M. (2010). Anti-inflammatory effects of resveratrol, curcumin and simvastatin in acute small intestinal inflammation. PLoS ONE.

[62] Heimesaat M.M., Alutis M., Grundmann U., Fischer A., Tegtmeyer N., Bohm M., Kuhl A.A., Gobel U.B., Backert S., Bereswill S. (2014). The role of serine protease HtrA in acute ulcerative enterocolitis and extra-intestinal immune responses during *Campylobacter jejuni* infection of gnotobiotic IL-10 deficient mice. Front. Cell. Infect. Microbiol..

[63] Alutis M.E., Grundmann U., Fischer A., Hagen U., Kuhl A.A., Gobel U.B., Bereswill S., Heimesaat M.M. (2015). The Role of Gelatinases in *Campylobacter Jejuni* Infection of Gnotobiotic Mice. Eur. J. Microbiol. Immunol..

[64] Alutis M.E., Grundmann U., Hagen U., Fischer A., Kuhl A.A., Gobel U.B., Bereswill S., Heimesaat M.M. (2015). Matrix Metalloproteinase-2 Mediates Intestinal Immunopathogenesis in *Campylobacter Jejuni*-Infected Infant Mice. Eur. J. Microbiol. Immunol..

[65] Heimesaat M.M., Lugert R., Fischer A., Alutis M., Kuhl A.A., Zautner A.E., Tareen A.M., Gobel U.B., Bereswill S. (2014). Impact of *Campylobacter jejuni* cj0268c knockout mutation on intestinal colonization, translocation, and induction of immunopathology in gnotobiotic IL-10 deficient mice. PLoS ONE.

[66] Heimesaat M.M., Nogai A., Bereswill S., Plickert R., Fischer A., Loddenkemper C., Steinhoff U., Tchaptchet S., Thiel E., Freudenberg M.A. (2010). MyD88/TLR9 mediated immunopathology and gut microbiota dynamics in a novel murine model of intestinal graft-versus-host disease. Gut.

[67] Bryan N.S., Grisham M.B. (2007). Methods to detect nitric oxide and its metabolites in biological samples. Free Radic. Biol. Med..

