# Supplementary figures and images for: Pituitary Adenylate Cyclase-Activating Polypeptide Alleviates Intestinal, Extra-Intestinal and Systemic Inflammatory Responses during Acute Campylobacter jejuni-induced Enterocolitis in Mice
Source: Pathogens. 2020 Sep 30;9(10):805. doi: 10.3390/pathogens9100805 (PMC7650764; doi:10.3390/pathogens9100805)

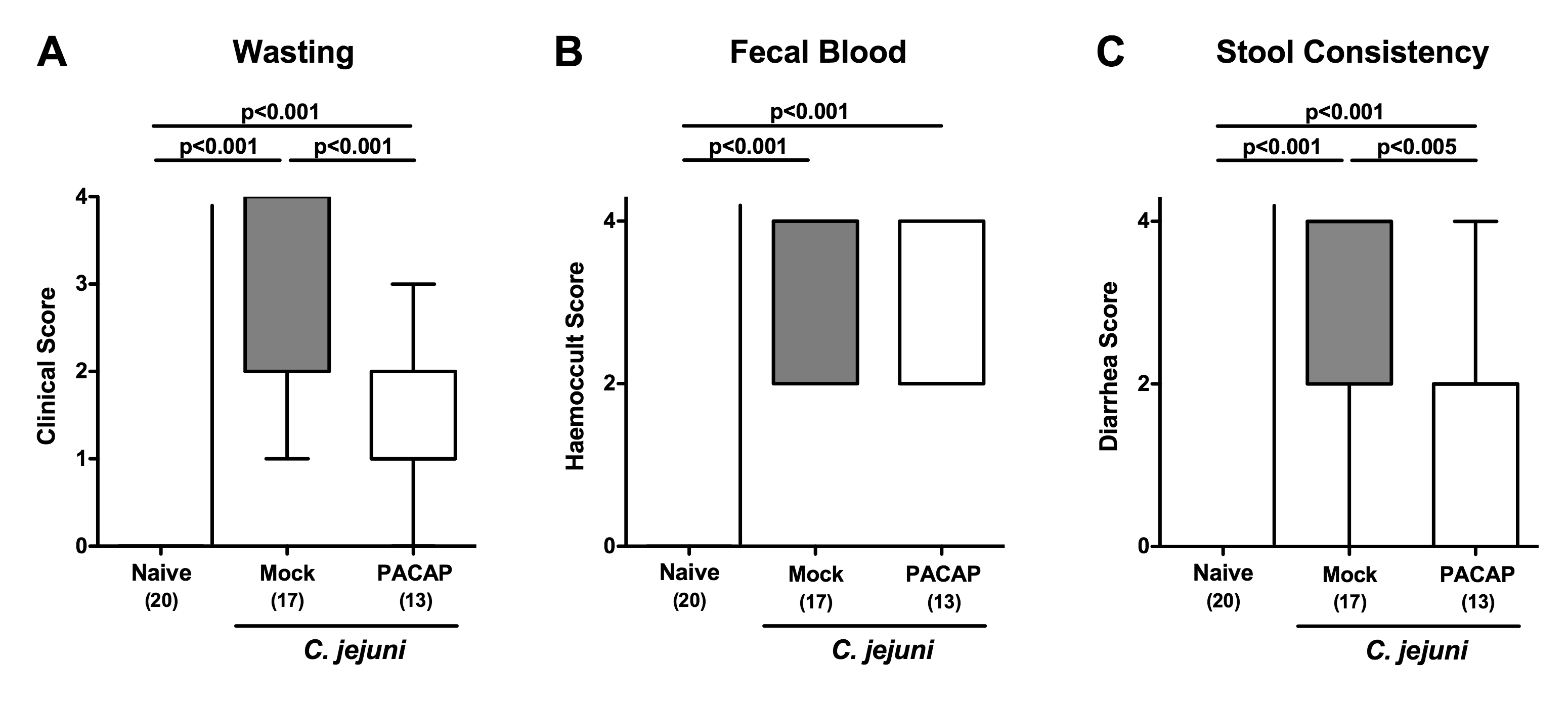

Supplement: Supplementary file 1 [file pathogens-09-00805-s001.zip › pathogens-870038-supplementary.tiff]
